# The Dangers Arising from Public Funerals

**Published:** 1891-01

**Authors:** 


THE DANGERS ARISING FROM PUBLIC FUNERALS of those who have died of Contagious Diseases. Circular No. 29, State Board of Health of Penna.

This circular is a protest against this class of funerals. It is especially directed to physicians, clergymen, and undertakers.

For copies of these circulars address the Secretary, Dr. Benjamin Lee, 1532 Pine Street, Philadelphia.

